# Mitofusin 2-Deficiency Suppresses Cell Proliferation through Disturbance of Autophagy

**DOI:** 10.1371/journal.pone.0121328

**Published:** 2015-03-17

**Authors:** Yanhong Ding, Han Gao, Lifang Zhao, Xian Wang, Ming Zheng

**Affiliations:** Department of Physiology and Pathophysiology, Health Science Center, Peking University, Beijing, China; University of Alabama at Birmingham, UNITED STATES

## Abstract

Mitofusin2 (Mfn2), a mitochondrial outer membrane protein serving primarily as a mitochondrial fusion protein, has multiple functions in regulating cell biological processes. Defects of Mfn2 were found in diabetes, obesity, and neurodegenerative diseases. In the present study, we found that knockdown of Mfn2 by shRNA led to impaired autophagic degradation, inhibited mitochondrial oxygen consumption rate and cell glycolysis, reduced ATP production, and suppressed cell proliferation. Inhibition of autophagic degradation mimicked Mfn2-deficiency mediated cell proliferation suppression, while enhancement of autophagosome maturation restored the suppressed cell proliferation by Mfn2-deficiency. Thus, our findings revealed the role of Mfn2 in regulating cell proliferation and mitochondrial metabolism, and shed new light on understanding the mechanisms of Mfn2 deficiency related diseases.

## Introduction

Mitochondria are important and complex organelles with essential functions in eukaryotic cells. As cellular “power engines”, mitochondria provide adenosine triphosphate (ATP) for cells through oxidative phosphorylation[1]. Mitochondria are also the main source of cellular ROS production[2], and participate in the regulation of local calcium levels[3,4]. Both ROS and calcium signals are actively involved in multiple cellular physiological or pathological events[5,6,7,8]. Moreover, it has been shown that mitochondria play a central role in controlling cell survival and death[9,10,11]. Thus, strictly quality control of mitochondria is of great importance for the maintenance of cellular homeostasis. Mitochondrial quality control is a process including the exchange of mitochondrial components through mitochondrial fusion and fission, and removal of the dysfunctional mitochondrion through autophagy or mitophagy. Defects of mitochondrial fusion and fission or impairment of mitophagy has been linked with numerous diseases such as Alzheimer’s disease, heart failure, and diabetes[12,13,14,15,16,17].

Mitofusin 2 (Mfn2) was originally identified as one of mitochondrial proteins mediating fusion of the mitochondrial outer membrane. Recently, it has been reported that Mfn2 also localizes on endoplasmic reticulum membrane and bridges the juxtaposition between endoplasmic reticulum and mitochondria thus regulating the local calcium concentration[18,19]. In fact, in addition to meditating the membrane fusion between organelles, Mfn2 plays multiple roles in various vital cellular processes including regulation of cell proliferation and cell survival/death, maintenance of mitochondrial DNA stability, and more recently, the regulation of ER stress and autophagy[20,21,22,23,24]. Mutations of Mfn2 are causally linked with autosomal dominant neurodegenerative disease Charcot-Marie-Tooth type 2A[25,26], obesity and type 2 diabetes[27]. We previously found that overexpression of Mfn2 led to cardiomyocyte apoptosis through the suppression of Akt activation in a mitochondrial fusion independent manner[28], while unexpectedly, cardiac deficiency of Mfn2 induced impaired autophagic degradation and cardiac dysfunction[23]. It has been reported that Mfn2 level was decreased in proliferative smooth muscle cells and hypertrophic cardiomyocytes, overexpression of Mfn2 suppressed VSMC proliferation through inhibition of ERK pathway and subsequent arrest of cell cycle [20,28], however, if deficiency of Mfn2 *per se* directly regulates cell proliferation has not yet been defined.

In the present study, we found that knockdown of Mfn2 inhibited, and re-expression of Mfn2 restored, HeLa cell proliferation. While down-regulation of Mfn2 resulted in impaired autophagic degradation and decreased mitochondrial and cellular metabolism, disturbance of autophagic degradation *per se* inhibited mitochondrial metabolism, ATP production as well as cell proliferation, suggesting that down-regulation of Mfn2 inhibits cell proliferation through impairment of autophagic process and mitochondrial metabolism. Thus, our findings illustrated the role of Mfn2 in regulating cell proliferation, thus shed new light on understanding mechanisms of Mfn2 deficiency related diseases.

## Materials and Methods

### Cell viability assay

HeLa cells or a human smooth muscle cell line (T/G HA-VSMC) [29] were seeded in a 96-well plate. After serum starvation for 24 hours to achieve mitogenic quiescence, adenovirus containing scramble RNA or Mfn2 shRNA[23] was infected into cells, at an MOI of 50, as control or Mfn2 knockdown, respectively. In subset experiments, cells were transfected with scramble RNA, Rab7 siRNA1 (5’-CGGUUCCAGUCUCUCGGUG-3’) or siRNA2 (5’-GAGCUGACUUUCUGACCAA-3’), or treated with Bafilomycin A1, 3-Methyladenine (3-MA) or NH4Cl. At time points as indicated, enzyme-based MTT assay or Cell Counting Kit-8 assay (CCK8, Dojindo Molecular Technologies) were performed to evaluate cell proliferation following the instruction of the manufacturer. Briefly, for MTT measurement, cells were cultured with 3-(4,5–Dimethylthiazol-2-yl)-2,5-diphenyltetrazolium bromide (MTT from Sigma) for 4 hours before measurement, then the supernatant was discarded and 100μl DMSO (Sigma) was added into each well. After gently shaking the plate in darkness for 10 minutes, the absorbance at 490 nm was measured with microplate absorbance reader (Bio-Rad). Each count was an average of three repeats and each data point was the average of at least three experiments.

Cell Counting Kit-8 assay was performed by using a tetrazolium salt, WST-8, which produces soluble WST-8 formazan. Briefly, at 2 hours before each indicated time point, 10μl of the CCK-8 solution was added to each well containing 100ul DMEM, the plate was then read by microplate absorbance reader and absorbance at 450 nm was recorded. Each count was an average of three repeats and each data point was the average of at least three experiments. All the data was normalized to the control group.

### Western blot analysis

Proteins were extracted from HeLa cells, subjected to 10% sodium dodecyl sulfate polyacrylamide gel electrophoresis, and then transferred to PVDF membranes. The membranes were probed with indicated primary antibodies at 4°C overnight (anti-LC3A/B and anti-Rab7 from CST, anti-Mfn2 from abnova, anti-Mfn1 from Santa Cruz Biotechnology and anti-GAPDH from Santa Cruz Biotechnology), then incubated with secondary antibodies (IRDye 700 or 800-conjugated anti-mouse or anti-rabbit IgG from Rockland Inc) for 1 hour at room temperature. Immunoblots were detected using the Odyssey infrared imaging system (LI-COR Biosciences, Lincoln, NE).

### Cell cycle and apoptosis assay by flow cytometry

After synchronized by serum-free starvation for 24 hours and infected with adenovirus for 24h hours, HeLa cells were then harvested and stained with propidium iodide using a Cycle TEST PLUS DNA Reagent Kit (Becton Dickinson, USA). Cell cycles were analyzed using flow cytometry with a FACScan (Becton Dickinson, USA).

Cell apoptosis was detected using an Annexin V-fluorescein isothiocyanate (FITC)/propidium iodide (PI) apoptosis detection kit (Invitrogen, USA) following the manufacturer's protocol. Briefly, cells were collected, centrifuged, and re-suspended in 500μl of 1×binding buffer in tubes. After added with Annexin V-FITC and PI, tubes were incubated darkly at room temperature for 15 minutes. Cell apoptosis assay was performed immediately on flow cytometry. Each experiment was performed at least three times.

### Confocal microscopy assay

For autophagic degradation assay, HeLa cells (1×10^5^) infected with adenovirus containing scramble or Mfn2 shRNA were co-transfected with a GFP-mRFP-LC3 expression plasmid by using the Lipofectamine 2000 (Invitrogen). 24 hours after transfection, cells were starved with serum free DMEM for 36 hours, and the GFP and mRFP fluorescent signals were detected using a confocal microscope (Leica, Bannockburn, IL), by exciting at 488nm and 561nm, and collecting emission at 505–530nm and >560 nm for GPF and mRFP, respectively.

### Plasmid construction and transfection

The cDNA with the full-length ORF of Tom1 (NM_005488.2) and Lamp2a (NM_002294.2) was amplified by PCR with following primers 5’-GCTCTAGAATGGACTTTCTCCTGGGGAAC-3’ (forward) and 5’- GCGGATCCTCATAAGGCAAACAGCATGTCATC—3’ (reverse) for Tom1; 5’-GGGGTACCAATGGTGTGCTTCCGCCTCTT-3’ (forward) and 5’-GCGGATCCCTAAAATTGCTCATATCCAGCAT-3’ (reverse) for Lamp2a. cDNAs were then cloned into the p3XFLAG-CMV-7.1 expression vector (Sigma) to produce Flag-tagged Tom1(Pcmv-Tom1) and Flag-tagged Lamp2a (Pcmv-Lamp2a) plasmids. Cells cultured in 6-well plates were transfected with plasmids by the Lipofectamine 2000 Transfection Reagent method (Invitrogen) and harvested for further experiments at time points as indicated. Primers for human Tom1 (sense: GGCATCTTTGGGACCTTC; antisense: TCTCCAGTGGGACAGCG) and Lamp2a (sense: GGCACCCACCATACA; antisense: GGCTGAACCCTTAGATC) were used for real-time PCR to confirm the gene expression.

### Mitochondrial respirometry and cell glycolysis assays

Respirometry of intact HeLa cells was performed using an XF24 Extracellular Flux Analyzer (Seahorse Biosciences). Cells were seeded at a density of 5X10^4^ cells/well in 24-well XF microplates, cultured with DMEM containing 10% FBS for 24 hours. One hour before initiation of measurements, medium was replaced with XF base medium supplemented with 25mM glucose and 2mM pyruvate. After 1 hour incubation in a CO_2_-free incubator at 37°C to allow temperature and pH equilibration, baseline oxygen consumption rate (OCR) was measured, then followed by injections sequentially with oligomycin (1 μM) to measure the ATP linked OCR, oxidative phosphorylation uncoupler FCCP (0.2 μM) to determine maximal respiration, and rotenone (1 μM) and antimycin A (1 μM) to determine the non-mitochondrial respiration. Experimental treatments were performed on 3 or 4 wells of each plate as technical replicates, and each experiment had at least 3 replicates. OCR was normalized by the amount of 100μg cellular protein in each well.

For cell glycolysis measurement, HeLa cells seeded in XF24 microplates as described above were cultured in XF base medium supplemented with 2mM L-glutamine in CO_2_-free incubator for 1 hour. After three baseline extracellular acidification rate (ECAR) measurement, cells were added sequentially with glucose (10mM) to measure the glucose metabolism, oligomycin (1μM) to measure the glycolytic capacity, and 2-deoxy-D-glucose (2-DG) (100mM) to measure the non-glycolytic acidification. Experimental treatments were performed on 3 or 4 wells of each plate as technical replicates and each experiment had at least 3 biological replicates. ECAR was normalized by the amount of 100μg protein in each well.

### ATP measurement

Intracellular ATP was measured by the luciferin/luciferase method using an ATP-Lite Assay Kit (Vigorous Biotechnology) following manufacturer's instruction. Briefly, HeLa cells were washed twice with ice-cold PBS, and lysed with lysis buffer. Cell lysis was centrifuged at 12000 rpm/min for 1 minute and supernatant was incubated with freshly prepared ATP assay mix and ATP releasing reagents, and then subjected to bioluminescent detection. The ATP level was presented as percentage to the untreated control group.

### Statistics

Data are presented as mean ± SEM. Unpaired t test with repeated-measures was applied, when appropriate, to determine the statistical significance of differences. P <0.05 was considered statistically significant.

## Results

### Mfn2 deficiency inhibits cell proliferation

**Fig 1:**
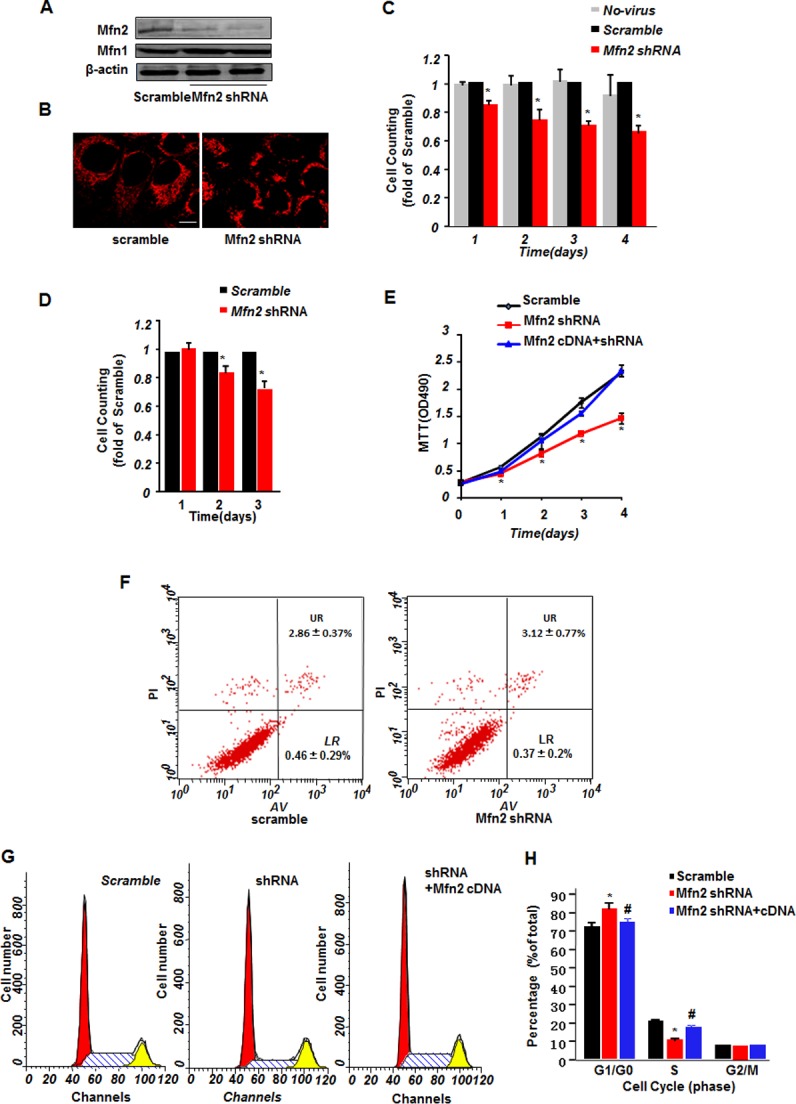
Mfn2 knockdown inhibits cell proliferation. (**A**) Western blotting showing Mfn2 and Mfn1 protein levels in HeLa cells infected with adenovirus containing scrambled RNA or two sets of Mfn2 shRNAs. β-actin was used as protein loading control. (**B**) Confocal imagings of HeLa cells stained with mitoTrackor showing mitochondrial morphology. Scale bar: 10 μm. (**C**) Cell counting kit-8 (CCK8) assay of HeLa cells without infection or infected with adenovirus containing scrambled RNA or Mfn2 shRNA. (**D**) CCK8 assay of T/G HA-VSMC cells infected with adenovirus containing scrambled RNA or Mfn2 shRNA at time point as indicated after infection. (**E**) 3-(4,5–dimethylthiazol-2-yl)-2,5-diphenyltetrazolium bromide (MTT) assay of HeLa cells infected with scrambled RNA, Mfn2 shRNA, or Mfn2 shRNA co-infected with Mfn2 cDNA. (**F**) Cell apoptosis assay by flow cytometry in HeLa cells infected with scrambled RNA or Mfn2 shRNA. (**G**) Fluorescence activated cell sorting analysis by flow cytometry to measure cell cycle distribution in HeLa cells infected with scrambled RNA, Mfn2 shRNA, or Mfn2 shRNA co-infected with Mfn2 cDNA, and (**H**) the histogram plot. n = 3–6 independent experiments for each group. *, p<0.05 versus scramble control.

To determine the effect of Mfn2 loss-of-function on cell proliferation, we infected HeLa cells with adenovirus containing short hairpin RNA of Mfn2 (Mfn2 shRNA) to knockdown Mfn2, or with adenovirus containing scramble RNA as control. Cells infected with Mfn2 shRNA for 48 hours significantly decreased Mfn2 protein level to about 36% of that in the scramble control cells, as indicated by western blot analysis (Fig. 1A), whereas the protein level of its homologue mitofusin 1(Mfn1), another outer mitochondrial membrane fusion protein, was not altered by Mfn2 shRNA. Down-regulation of Mfn2 led to mitochondrial fragmentation as compared with the thread-shaped mitochondria in scramble control cells (Fig. 1B & S1 Fig.), in consistent with previous reports that deficiency of Mfn2 causes mitochondrial fragmentation due to decreased mitochondrial fusion[27,30]. Surprisingly, in contrast to our expectation, cells infected with Mfn2 shRNA showed inhibited cell proliferation rate at as early as 1 day after Mfn2 shRNA infection, to 86.7±2.16% of cells without adenovirus infection or 85.5±2.09% of cells infected with scramble RNA, and to 74.7±7.43%, 70.8±3.09%, and 65.8±4.69% of scramble RNA control cells at day 2, day 3, and day 4 after Mfn2 shRNA infection, respectively, as detected by CCK8, a cell counting kit-8 assay (Fig. 1C). Unlike the inhibitory function of Mfn2 knockdown on cell proliferation, knockdown of Mfn1 by siRNA showed no effect on cell proliferation in spite of the fragmented mitochondria (S2 Fig.). Similar growth suppressive effect by Mfn2 knockdown was detected in a human smooth muscle cell line T/G HA-VSMC (Fig. 1D). We also employed another cell proliferative assay 3-(4,5–dimethylthiazol-2-yl)-2,5-diphenyltetrazolium bromide (MTT) to confirm the role of Mfn2 in cell proliferation. Mfn2 knockdown resulted in decreased MTT absorbance to 78±3.17%, 73.1±5.78%, 67.6±1.2%, and 63.8±9.56% of that in scramble RNA cells at day 1, day 2, day 3, and day 4 after infection, respectively, and this blunted cell growth by Mfn2 knockdown was totally restored by co-expression of Mfn2 cDNA with Mfn2 shRNA (Fig. 1E), to a protein level comparable of that in scramble control cells (data not shown). Direct counting cell numbers showed similar suppressive results in Mfn2 shRNA transfected cells (S3 Fig.). Our data here shown that Mfn2-deficiency inhibits HeLa cell and smooth muscle proliferation.

The decreased CCK8 cell counting or MTT absorbance might be caused either by suppression of cell proliferation, or by reduction of cell viability. Although no difference in cell morphology was detected with transmitted light microscope (data not shown), we further clarified if the Mfn2 deficiency-mediated growth suppression was due to an increase of apoptotic cells by flow cytometry using annexin V-fluorescein isothiocyanate (FITC)/propidium iodide (PI) apoptosis detection kit. No difference between scramble control and Mfn2 shRNA cells was found (Fig. 1F), with a LR (LR quadrant indicates the percentage of early apoptotic cells to Alexa 488-stained cells) of 0.37±0.2% in Mfn2 shRNA and 0.46±0.29% in scramble control, and a UR (UR quadrant indicates the percentage of late apoptotic cells to Alexa 488- and propidium iodide-stained cells) of 3.12±0.77% in Mfn2 shRNA cells and 2.86±0.37% in scramble control. Hochest 33342 staining assay showed similar results (S3 Fig.), excluding the apoptotic effect mediated by Mfn2 deficiency. Furthermore, we examined the cell cycle distribution by fluorescence activated cell sorting analysis with flow cytometry, and found that, whereas both cells infected with Mfn2 shRNA and with scramble RNA were mainly distributed in G1/G0 phase, with 81.85±3.6% for Mfn2 shRNA and 72.36±2.35% for scramble RNA, Mfn2 shRNA infected HeLa cells showed less distribution of 10.7±0.98% in S phase, as compared with 20.63±1.25% for scramble RNA controls. Co-expression of Mfn2 cDNA with Mfn2 shRNA largely increased the cells in S phase to17.77±0.79% (Fig. 1G & 1H). Altogether, our data suggest that deficiency of Mfn2 inhibits HeLa cell proliferation rather than induces apoptosis.

### Mfn2-deficiency suppresses cell proliferation through impairing autophagic degradation

**Fig 2:**
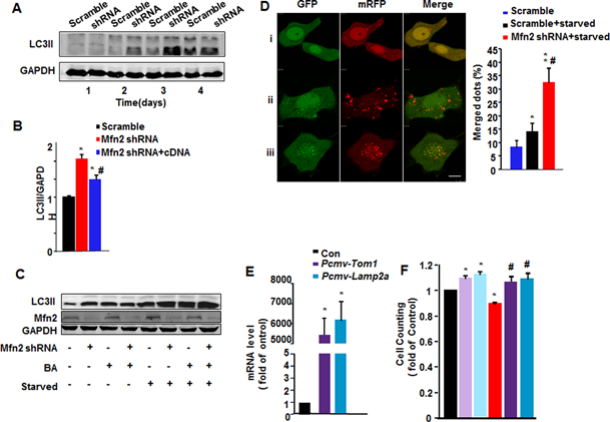
Mfn2 knockdown impairs autophagic degradation. (**A**) Increased LC3-II level in Mfn2 knockdown HeLa cells by Western blot. n = 4 independent experiments for each group. (**B**) LC3-II:GAPDH ratios in HeLa cells infected with scrambled RNA, Mfn2 shRNA, or Mfn2 shRNA with Mfn2 cDNA. n = 3 independent experiments for each group. *, p<0.05 versus scramble control; ^#^, p<0.05 versus Mfn2 shRNA group. (**C**) Western blot showing LC3-II levels in scramble and Mfn2 shRNA cells with or without starvation treatment in the presence or absence of lysosomal inhibitor Bafilomycin A1 (BA). (**D**) Confocal images of HeLa cells transfected with GFP-mRFP-LC3 plasmid, and co-infected with adenovirus containing scrambled RNA or Mfn2 shRNA, with or without starvation for 36 hours (left), and percentages of merged green and red LC3 dots to total LC3 dots. Scale bar: 10 μm. n = 20 cells for each group from 3 independent experiments. *, p<0.05 & **, p<0.01 versus scramble control; ^#^, p<0.05 versus scramble starvation group. (**E**) mRNA level of Tom1 and Lamp2a by real-time PCR in HeLa cells transfected with Pcmv-Tom1 or Pcmv-Lamp2a plasmids. n = 3 independent experiments. (**F**) Fold changes of cell counting by CCK8 in HeLa cells infected with adenovirus containing scrambled RNA co-transfected with Pcmv vector or Pcmv-Tom1, Pcmv-Lamp2a, and Mfn2 shRNA, Mfn2 shRNA co-transfected with Pcmv-Tom1 or Pcmv-Lamp2a. n = 3 independent experiments. *, p<0.05 versus control; ^#^, p<0.05 versus Mfn2 shRNA only group.

We previously found that depletion of Mfn2 led to accumulation of autophagosomes through the impairment of autophagic degradation in heart [23]. Here we further tested if deficiency of Mfn2 in HeLa cells regulates autophagy. The cells infected with Mfn2 shRNA showed increased lipidated and autophagosome-associated form of LC3 (LC3-II), the hallmark of autophagic processing at the molecular level, from the second day after shRNA infection, as compared with scramble controls (Fig. 2A). The average 1.6-fold increase of LC3-II protein level over the scramble control could be significantly diminished, though not completely blocked, by co-expression of Mfn2 cDNA with shRNA, to 82.5±3.91% of that with only Mfn2 shRNA infection (Fig. 2B). The higher LC3-II level in the presence of Mfn2 shRNA was further increased by starvation. However, bafilomycin A1, an autophagosome-lysosome fusion inhibitor, incrased LC3-II in scramble control cells with or without starvation treatment, failed to further enhance Mfn2 deficiency-induced increase of LC3-II (Fig. 2C), supporting our previous finding in cardiomyocytes that Mfn2 regulates autophagic process at the step of autophagic degradation. Moreover, a GFP-mRFP-LC3 plasmid was employed to visualize the autophagic degradation process [31]. LC3 puncta shows both green and red fluorescence (yellow when merged) before fusing with lysosome, but only red signal after the fusion due to the loss of GFP fluorescence in acidic conditions in lysosomes. In starved scramble HeLa cells, there are more LC3-positive autophagic vacuoles as compared with un-starved cells, however, most LC3 vacuoles displayed only red signals, the percentages of yellow puncta are 14.2±3.19% in starved scramble cells and 8.33±2.49% in scramble cells without starvation (Fig. 2D). In sharp contrast, starvation of Mfn2 shRNA cells caused increased LC3 puncta with incremental yellow fluorescent signals to a percentage of 32.6±8.8% (Fig. 2D), confirming the impaired autophagic degradation in Mfn2-deficiency HeLa cells.

The question raised here is whether Mfn2 deficiency-mediated impairment of autophagic degradation is associated with its suppression of cell proliferation. To answer it, we at first tested if impairment of autophagic degradation is sufficient to inhibit cell proliferation. HeLa cells were treated with autophagic degradation inhibitors Bafilomycin A1, 3-Methyladenine (3-MA), or NH4Cl (S4 Fig.), or transfected with autophagosome maturation related protein Rab7 siRNA to mimic the interruption of autophagic degradation (S4 Fig.). In consistent with the suppression of cell growth by Mfn2 deficiency, all three autophagic degradation inhibitors and two sets of Rab shRNAs significantly inhibited cell proliferation, as indicated by cell counting kit assay (S4 Fig.), indicating that Mfn2 deficiency-induced autophagic defects are causally related with its suppression of cell proliferation.

We then tested if increased autophagic degradation could restore the Mfn2 deficiency-induced suppression of cell proliferation. Two proteins, endosomal protein Tom1 and lysosome receptor Lamp2a, have been reported to mediate autophagosome-lysosome fusion, thus playing important roles in autophagosome maturation [32,33,34]. We here transiently over-expressed Tom1 or Lamp2a in control and Mfn2 shRNA infected HeLa cells by transfection of plasmids containing Tom1 cDNA or Lamp2a cDNA (Fig. 2E). Cells transfected with Pcmv-GFP vector was used as control cells, and the co-transfection of Pcmv-GFP plasmid with Mfn2 shRNA did not alter the knockdown efficiency (S5 Fig.). While knockdown of Mfn2 suppressed cell growth to 89.1±1.52%, and over-expression of Tom1 or Lamp2a accelerated cell growth to 108.9±2.3% and 111.6±2.5% of control cells infected with Pcmv vector, as assayed by CCK8 cell counting kit, co-expression of either Tom1 or Lamp2a with Mfn2 shRNA significantly restored the suppressed cell growth by Mfn2-deficiency, to 106.2±3.12% and 109.2±4.6% respectively, as compared with Pcmv control cells (Fig. 2F). Collectively, our data here demonstrated that Mfn2 deficiency-induced impairment of autophagic degradation contributes to the suppression of cell growth.

### Mfn2-deficiency disturbs mitochondrial respiration, glycolysis, and ATP production

**Fig 3:**
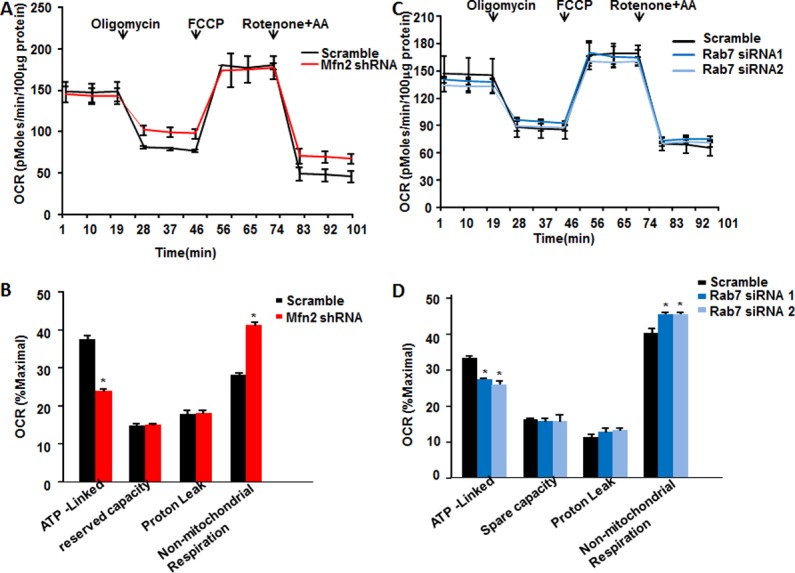
Cell oxygen consumption rates by Mfn2 knockdown or inhibition of autophagic degradation. Traces of oxygen consumption rates (OCR) of HeLa cells infected with (**A**) adenovirus containing scrambled RNA or Mfn2 shRNA or (**C**) scrambled RNA, Rab7 siRNA1, or Rab7 siRNA2, as measured with the XF24 metabolic analyzer by sequential, in port additions of mitochondrial effectors at time points indicated by downward arrows. (**B**) (**D**) Percentages of ATP-linked OCR, proton-leak OCR, reserve capacity, and non-mitochondrial OCR to maximal OCR. n = 3 independent experiments for each group. *, p<0.05 versus control.

Since mitochondria are organelles where mitochondrial respiration and oxidative phosphorylation occur, we then examined if Mfn2 deficiency and the consequently impaired autophagic degradation affect mitochondrial oxidative phosphorylation by measuring oxygen consumption rate (OCR). The measurement of the mitochondrial profile is performed by sequentially adding ATP synthase inhibitor oligomycin, un-coupler FCCP, and the combination of electron transport chain inhibitors rotenone with antimycin A. The optimal doses were determined by dose response curves (S6 Fig.). The rates of oxygen consumption which attribute to ATP production, maximal respiration, and proton leak, were monitored. We first determined the basal mitochondrial functions of scramble control and Mfn2 shRNA infected HeLa cells in medium with 25mM glucose and 2mM pyruvate. We found that oligomycin caused less reduction of the mitochondrial OCR, to 24.1±1.31% of the maximal OCR, in Mfn2 deficiency cells, as compared with the reduction of 37.9±3.12% of maximal in scramble control cells, indicating a decreased ATP synthesis-linked OCR by Mfn2 deficiency. Moreover, the non-mitochondrial OCR increased from 28.4±0.95% in scramble control cells to 42.2±2.97% in mfn2-deficiency cells, whereas proton leak associated OCR and mitochondrial reserved capacity did not change (Fig. 3A & 3B). Similarly, HeLa cells transfected with Rab7 siRNAs showed less ATP synthesis-linked OCR and increased non-mitochondrial OCR, but without changing proton leak-linked OCR and mitochondrial reserve capacity (Fig. 3C & 3D). The ATP synthesis-linked OCR was decreased from 33.3±1.97% in scramble control cells to 26.7±2.07% in Rab7 siRNA 1 transfected cells and 25.8±2.56% in Rab7 siRNA 2 cells respectively while non-mitochondrial OCR was increased from 40.1±2.07% in scramble control cells to 45.2±0.97% in Rab7 siRNA 1 transfected cells and 45.2±0.71% in Rab7 siRNA 2 cells respectively (Fig. 3D), in comparable levels to that in Mfn2-deficiency cells. Similar results were obtained in cells treated with NH4Cl to interrupt the degradation of autophagy (data not shown). Thus, here our data suggest that defects of Mfn2 disturb mitochondrial respiration due to the impaired autophagic degradation.

**Fig 4:**
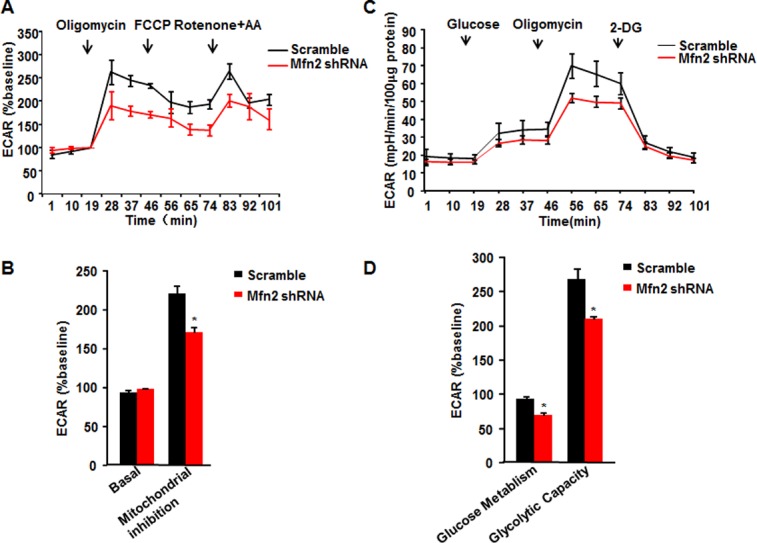
Inhibited glycolysis by Mfn2 knockdown. (**A**) Traces of extracellular acidification rates (ECAR) of HeLa cells in response to mitochondrial inhibitors. (**B**) Average data of basal ECAR and that in the presence of mitochondrial inhibitors as in **A**. (**C**) ECAR of HeLa in response to glucose, oligomycin, and 2-deoxy-D-glucose (2-DG). (**D**) ECAR showing glucose metabolism and glycolytic capacity. n = 3 independent experiments for each group. *, p<0.05 versus scramble control.

Simultaneously, we measured the rate of extracellular acidification (ECAR) in the aforementioned set of mitochondrial metabolic profile, as assigned to glycolysis. Surprisingly, while Mfn2 deficiency did not alter basal ECAR, it significantly suppressed ECAR in response to treatments of mitochondrial ETC manipulators (Fig. 4A), to an average level of 77.3±3.5% of that in scramble control cells (Fig. 4B), indicating the inhibition of glycolysis by Mfn2 deficiency. Likely, treatment with NH4Cl or knockdown of Rab7 by both sets of siRNAs significantly inhibited ECAR in response to mitochondrial ETC manipulators (S7 Fig.). To further confirm the effect of Mfn2 knockdown on cell glycolysis, we measured cell response to glucose metabolism. Consistently, Mfn2 knockdown inhibited ECAR of cells in response to glucose, to 74.5±3.2% comparing with scramble control cells. Also, ECAR in response to oligomycin was inhibited to 78.2±5.6% of the control cells, while ECAR in response to 2-deoxy-D-glucose (2-DG) was not changed, indicating that down-regulation of Mfn2 inhibited cell glycolytic metabolism (Fig. 4C&4D). Together, these data suggest that Mfn2 regulates mitochondrial respiration and cell glycolysis, at least partly through mediating autophagic degradation.

**Fig 5:**
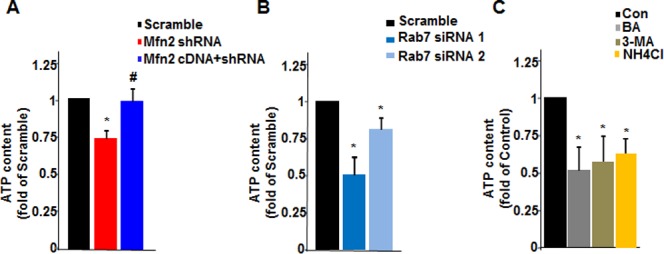
Reduced ATP production by Mfn2 knockdown or inhibition of autophagic degradation. Intracellular ATP production of HeLa cells (**A**) infected with scrambled RNA, Mfn2 shRNA, or Mfn2 shRNA co-infected with Mfn2 cDNA, (**B**) transfected with scrambled RNA, Rab7 siRNA1, or Rab7 siRNA2, or (**C**) treated with autophagic degradation inhibitors Bafilomycin A1, 3-Methyladenine (3-MA), or NH4Cl. n = 4 independent experiments for each group. *, p<0.05 versus control cells; #, p<0.05 versus Mfn2 shRNA group.

Because mitochondrial oxidative phosphorylation and cell glycolysis are main sources of cellular ATP, we next measured ATP production. Knockdown of Mfn2 in HeLa cells caused decreased ATP production to 73.7±3.6% of that in scramble control cells, and co-expression of Mfn2 with shRNA restored the ATP production to 98.3±4.8% (Fig. 5A), suggestive of the depressed energy production by Mfn2 deficiency. Then, we further investigated the role of impaired autophagy in ATP production. Knockdown of Rab7 by Rab7 siRNA1 and siRNA2 reduced ATP production to 50±6.2% and 83.5±4.9% of scramble control cells, respectively (Fig. 5B). Furthermore, disturbing autophagic degradation by Bafilomycin A1, 3-Methyladenine, or NH4Cl all led to the reduction of ATP production, to 51.4±7.5%, 57.2±7.6%, and 63.5±5.1% as compared with untreated control cells (Fig. 5C). Altogether, these findings suggest that Mfn2 deficiency-mediated autophagic impairment inhibits cell ATP production.

## Discussion

In the present study, we demonstrated the novel role of Mfn2 in regulating cell proliferation through mediating cell autophagy and bioenergy. Firstly, down-regulation of Mfn2 in HeLa cells or T/G HA-VSMC cells suppressed, while re-expression of Mfn2 restored, cell proliferation. Secondly, knockdown of Mfn2 in HeLa cells interrupted autophagosome-lysosome fusion. Whereas inhibiting autophagic degradation *per se* sufficiently suppressed HeLa cell proliferation, enhancing autophagosome maturation restored Mfn2 deficiency-mediated suppression of cell proliferation. Thirdly, down-regulation of Mfn2 or inhibition of autophagic degradation reduced mitochondrial respiration and cell glycolysis, and largely suppressed cellular ATP production, indicating that Mfn2 deficiency mediated impairment of autophagic degradation causes failure of bioenergenesis and consequently leads to suppression of cell proliferation.

We previously found that in cardiomyocytes Mfn2 protein level was increased during oxidative stress induced apoptosis, and overexpression of Mfn2 caused cardiomyocyte apoptosis through a mitochondria-dependent pathway[28]. In contrast to our findings, Parra V et al found that down-regulation of Mfn2 exacerbated ceramide-induced cardiomyocyte apoptosis[35]. The discrepancy of these findings implies that down-regulation or up-regulation of Mfn2 may mediate same cellular process through different pathways under different conditions[36]. In this context, we explored the effect of Mfn2 loss-of-function on cell proliferation. Surprisingly, while previous studies showed over-expression of Mfn2 inhibited smooth muscle cell proliferation and led to cardiomyocyte and smooth muscle cell apoptosis, here we found that down-regulation of Mfn2 similarly repressed T/G HA-VSMC cell and HeLa cell proliferation, although with different mechanisms. That Mfn2 gain-of-function and loss-of-function both caused repression of cell proliferation indicates the importance of balanced Mfn2 protein level in maintaining normal cell functions. Given that Mfn2 defect contributes to metabolic defects associated with obesity, neuropathy, as well as cardiac dysfunction[23,37,38], it is understandable that Mfn2-deficiency leads to the inhibition of cell proliferation. However, in contrast to our finding that down-regulation of Mfn2 repressed cell proliferation, a recent paper reported that Mfn2-null mouse embryonic fibroblast (MEF) and a Mfn2 knockdown clone of lymphoma cell line BJAB displayed increased cell proliferation through Ras-Raf-ERK pathway[39]. Indeed, we also observed increased growth rate and distinct cell shape of Mfn2-null MEF as compared with wild type MEF (data not shown), however, transient knockdown of Mfn2 by shRNA infection to HeLa cell or T/G HA-SMC suppressed cell growth as comparing with scramble control cells. The mechanisms underlying these disagreements need further investigation, however, different cell lines studied and times of Mfn2-knockdown or knockout in cells may contribute to the discrepancy.

Our previous study showed that cardiac Mfn2 in the heart is involved in the regulation of autophagy, the conserved process in all eukaryotes by self-degradating intracellular components and damaged organelles to maintain normal organelles function and nutrient restoration[23]. The present study found that the impaired autophagic degradation mediated by Mfn2 deficiency caused reduction of mitochondrial respiration, cell glycolysis and bioenergenesis. Importantly, enhancing the autophagosome maturation completely restored the suppressed cell proliferation by Mfn2-deficiency. Mfn2 may regulate cell metabolism from two aspects. The first one is that Mfn2 maintains mitochondrial respiration through directly affecting components of mitochondrial electron transport chain[37]. Deficiency of Mfn2 inhibited the expression of ETC complexes I/II/III/V[37] and caused neuropathy and obesity[25,26,40], this is in general agreement with our present results that Mfn2 knockdown reduced mitochondrial oxygen consumption rate. Another one is that Mfn2 mediates cellular metabolism through the regulation of autophagy. Although a large body of evidence demonstrates that autophagy serves a housekeeping function and provides internal nutrients to maintain cellular metabolism, it is still controversial about the roles of autophagy in cancer survival or death. While active autophagy suppresses tumorigenesis[41,42], autophagy also supports tumor survival and growth through distinct pathways[43,44]. Our present study found that blocking autophagic degradation *per se* inhibited the mitochondrial OCR and cell ECAR, and reduced cellular ATP production, in consistent with the suppressed mitochondrial and cellular metabolism mediated by deficiency of Mfn2.

In summary, our data have shown that Mfn2 deficiency inhibited cell proliferation through impaired autophagic process and subsequently disturbed cell metabolism. Our findings not only illustrate the role of Mfn2 in regulating cell proliferation, but also shed new light on understanding the mechanisms of Mfn2 deficiency related diseases.

## Supporting Information

S1 Fig Mitochondrial morphology. Confocal imagings of scramble and Mfn2 shRNA transfected HeLa cells stained with mitoTrackor showing mitochondrial morphology at time points after transfection as indicated.
(TIF)

S2 Fig Mfn1 knockdown has no effect on cell proliferation. (**A**) Confocal imagings of scramble (left) and Mfn1 siRNA (right) infected HeLa cells stained with mitoTrackor. (**B**) Cell counting kit-8 (CCK8) assay of scramble or Mfn1 siRNA infected HeLa cells at indicated time after infection.
(TIF)

S3 Fig Cell counting. (**A**) Cell number counting of scramble and Mfn2 shRNA transfected HeLa cells by a cytometer at indicated time point after transfection. (**B**) Apoptotic cell counting of Hela cells stained with Hoechst 33342 by fluorescence microscope.
(TIF)

S4 Fig Inhibition of autophagic degradation suppresses cell proliferation. (**A**) Relative fold changes of cell counting by CCK8 in HeLa cell treated with autophagic degradation inhibitors Bafilomycin A1, 3-Methyladenine (3-MA), or NH4Cl as comparing with cells treated with DMSO. n = 3 independent experiments for each group. (**B**) Rab7 protein levels by western blotting in HeLa cells transfected with scrambled RNA, Rab7 siRNA1, or Rab7 siRNA2. n = 3 independent experiments. (**C**) Fold changes of cell counting by CCK8 in HeLa cells transfected with Rab7 siRNA1, or Rab7 siRNA2 comparing with cells transfected with scrambled RNA. n = 3–5 independent experiments. *, p<0.05 versus control.
(TIF)

S5 Fig mRNA levels of Tom1, Lamp2a, and Mfn2. (**A**) mRNA level of Tom 1 by RT-PCR in scramble or Mfn2 shRNA transfected cells co-expressed with Pcmv-GFP or Pcmv-Tom1 plasmids. (**B**) mRNA level of Lamp2a by RT-PCR in scramble or Mfn2 shRNA transfected cells co-expressed with Pcmv-GFP or Pcmv-Lamp2a plasmids. (**C**) mRNA level of Mfn2 by RT-PCR in scramble or Mfn2 shRNA transfected cells co-expressed with Pcmv-GFP, Pcmv-Tom1, or Pcmv-Lamp2a plasmid. n = 3 independent experiments.
(TIF)

S6 Fig Dose response curves of the oxygen consumption rates of HeLa cells infected with scramble or Mfn2 shRNA in response to mitochondrial inhibitors Oligomycin (A), FCCP (B), and antimycin A +Rotenone. Data were presented as difference of OCR between cells with and without mitochondrial inhibitor stimulation. n = 3 independent experiments for each group.
(TIF)

S7 Fig Inhibited glycolysis by Rab7 knockdown. (**A**) Traces of extracellular acidification rates (ECAR) of HeLa cells in response to mitochondrial inhibitors. (**B**) Average data of basal and ECAR in the presence of mitochondrial inhibitors as in **A**. n = 3 independent experiments for each group. *, p<0.05 versus scramble control.
(TIF)

## References

[1] Paumard P, Vaillier J, Coulary B, Schaeffer J, Soubannier V, Mueller DM, et al The ATP synthase is involved in generating mitochondrial cristae morphology. The EMBO Journal. 2002; 21: 221–230. 11823415 10.1093/emboj/21.3.221 PMC125827

[2] Chandel NS, Maltepe E, Goldwasser E, Mathieu CE, Simon MC, Schumacker PT. Mitochondrial reactive oxygen species trigger hypoxia-induced transcription. Proc Natl Acad Sci U S A 1998; 95: 11715–11720. 9751731 10.1073/pnas.95.20.11715 PMC21706

[3] Deluca HF, Engstrom GW. Calcium uptake by rat kidney mitochondria. Proc Natl Acad Sci U S A 1961;47: 1744–1750. 13885269 10.1073/pnas.47.11.1744 PMC223205

[4] Vasington FD, Murphy JV. Ca ion uptake by rat kidney mitochondria and its dependence on respiration and phosphorylation. J Biol Chem 1962; 237: 2670–2677. 13925019

[5] Krieger C, Duchen MR. Mitochondria, Ca2+ and neurodegenerative disease. Eur J Pharmacol 2002; 447: 177–188. 12151010 10.1016/s0014-2999(02)01842-3

[6] Duchen MR. Mitochondria and calcium: from cell signalling to cell death. J Physiol 2000; 529 Pt 1: 57–68. 11080251 10.1111/j.1469-7793.2000.00057.x PMC2270168

[7] Rosenstock TR, Carvalho AC, Jurkiewicz A, Frussa-Filho R, Smaili SS. Mitochondrial calcium, oxidative stress and apoptosis in a neurodegenerative disease model induced by 3-nitropropionic acid. J Neurochem 2004; 88: 1220–1228. 15009678 10.1046/j.1471-4159.2003.02250.x

[8] Shigenaga MK, Hagen TM, Ames BN. Oxidative damage and mitochondrial decay in aging. Proc Natl Acad Sci U S A 1994; 91: 10771–10778. 7971961 10.1073/pnas.91.23.10771 PMC45108

[9] Kroemer G, Marino G, Levine B. Autophagy and the integrated stress response. Mol Cell 2010; 40: 280–293. 10.1016/j.molcel.2010.09.023 20965422 PMC3127250

[10] Vandenabeele P, Galluzzi L, Vanden Berghe T, Kroemer G. Molecular mechanisms of necroptosis: an ordered cellular explosion. Nat Rev Mol Cell Biol 2010; 11: 700–714. 10.1038/nrm2970 20823910

[11] Balaban RS, Nemoto S, Finkel T. Mitochondria, oxidants, and aging. Cell 2005; 120: 483–495. 15734681 10.1016/j.cell.2005.02.001

[12] Liesa M, Shirihai OS. Mitochondrial dynamics in the regulation of nutrient utilization and energy expenditure. Cell Metab 2013; 17: 491–506. 10.1016/j.cmet.2013.03.002 23562075 PMC5967396

[13] Liu L, Feng D, Chen G, Chen M, Zheng Q, Song P, et al Mitochondrial outer-membrane protein FUNDC1 mediates hypoxia-induced mitophagy in mammalian cells. Nat Cell Biol 2012; 14: 177–185. 10.1038/ncb2422 22267086

[14] Nixon RA. The role of autophagy in neurodegenerative disease. Nat Med 2013; 19: 983–997. 10.1038/nm.3232 23921753

[15] Osellame LD, Rahim AA, Hargreaves IP, Gegg ME, Richard-Londt A, Brandner S, et al Mitochondria and quality control defects in a mouse model of Gaucher disease—links to Parkinson's disease. Cell Metab 2013; 17: 941–953. 10.1016/j.cmet.2013.04.014 23707074 PMC3678026

[16] Youle RJ, Narendra DP. Mechanisms of mitophagy. Nat Rev Mol Cell Biol 2011; 12: 9–14. 10.1038/nrm3028 21179058 PMC4780047

[17] Gomes LC, Di Benedetto G, Scorrano L. During autophagy mitochondria elongate, are spared from degradation and sustain cell viability. Nat Cell Biol 2011; 13: 589–598. 10.1038/ncb2220 21478857 PMC3088644

[18] de Brito OM, Scorrano L. Mitofusin 2 tethers endoplasmic reticulum to mitochondria. Nature 2008; 456: 605–610. 10.1038/nature07534 19052620

[19] de Brito OM, Scorrano L. An intimate liaison: spatial organization of the endoplasmic reticulum-mitochondria relationship. EMBO J 2010; 29: 2715–2723. 10.1038/emboj.2010.177 20717141 PMC2924651

[20] Chen KH, Guo X, Ma D, Guo Y, Li Q, Yang D, et al Dysregulation of HSG triggers vascular proliferative disorders. Nat Cell Biol 2004; 6: 872–883. 15322553 10.1038/ncb1161

[21] Wang W, Cheng X, Lu J, Wei J, Fu G, Zhu F, et al Mitofusin-2 is a novel direct target of p53. Biochem Biophys Res Commun 2010; 400: 587–592. 10.1016/j.bbrc.2010.08.108 20804729

[22] Jin B, Fu G, Pan H, Cheng X, Zhou L, Lv J, et al Anti-tumour efficacy of mitofusin-2 in urinary bladder carcinoma. Med Oncol 2011; 28 Suppl 1: S373–380. 10.1007/s12032-010-9662-5 20803103

[23] Zhao T, Huang X, Han L, Wang X, Cheng H, Xiao R, et al Central role of mitofusin 2 in autophagosome-lysosome fusion in cardiomyocytes. J Biol Chem 2012; 287: 23615–23625. 10.1074/jbc.M112.379164 22619176 PMC3390636

[24] Chen H, Vermulst M, Wang YE, Chomyn A, Prolla TA, McCaffery JM, et al Mitochondrial fusion is required for mtDNA stability in skeletal muscle and tolerance of mtDNA mutations. Cell 2010; 141: 280–289. 10.1016/j.cell.2010.02.026 20403324 PMC2876819

[25] Baloh RH, Schmidt RE, Pestronk A, Milbrandt J. Altered axonal mitochondrial transport in the pathogenesis of Charcot-Marie-Tooth disease from mitofusin 2 mutations. J Neurosci 2007; 27: 422–430. 17215403 10.1523/JNEUROSCI.4798-06.2007 PMC6672077

[26] Cartoni R, Martinou JC. Role of mitofusin 2 mutations in the physiopathology of Charcot-Marie-Tooth disease type 2A. Exp Neurol 2009; 218: 268–273. 10.1016/j.expneurol.2009.05.003 19427854

[27] Sebastian D, Hernandez-Alvarez MI, Segales J, Sorianello E, Munoz JP, Sala D, et al Mitofusin 2 (Mfn2) links mitochondrial and endoplasmic reticulum function with insulin signaling and is essential for normal glucose homeostasis. Proc Natl Acad Sci U S A 2012; 109: 5523–5528. 10.1073/pnas.1108220109 22427360 PMC3325712

[28] Shen T, Zheng M, Cao C, Chen C, Tang J, Zhang W, et al Mitofusin-2 is a major determinant of oxidative stress-mediated heart muscle cell apoptosis. J Biol Chem 2007; 282: 23354–23361. 17562700 10.1074/jbc.M702657200

[29] Limor R, Kaplan M, Sawamura T, Sharon O, Keidar S, Weisinger G, et al Angiotensin II increases the expression of lectin-like oxidized low-density lipoprotein receptor-1 in human vascular smoothmuscle cells via a lipoxygenase-dependent pathway. Am J Hypentens 2005; 18: 299–307. 15797645 10.1016/j.amjhyper.2004.09.008

[30] Chen H, Detmer SA, Ewald AJ, Griffin EE, Fraser SE, Chan DC. Mitofusins Mfn1 and Mfn2 coordinately regulate mitochondrial fusion and are essential for embryonic development. J Cell Biol (2003) 160: 189–200. 12527753 10.1083/jcb.200211046 PMC2172648

[31] Chen D, Fan W, Lu Y, Ding X, Chen S, Zhong Q. A mammalian autophagosome maturation mechanism mediated by TECPR1 and the Atg12-Atg5 conjugate. Mol Cell 2012; 45:629–641. 10.1016/j.molcel.2011.12.036 22342342 PMC3299828

[32] Tumbarello DA, Waxse BJ, Arden SD, Bright NA, Kendrick-Jones J, Buss F. Autophagy receptors link myosin VI to autophagosomes to mediate Tom1-dependent autophagosome maturation and fusion with the lysosome. Nat Cell Biol 2012; 14: 1024–1035. 10.1038/ncb2589 23023224 PMC3472162

[33] Tumbarello DA, Kendrick-Jones J, Buss F. Myosin VI and its cargo adaptors—linking endocytosis and autophagy. J Cell Sci 2013; 126: 2561–2570. 10.1242/jcs.095554 23781020 PMC3687694

[34] Tanaka Y, Guhde G, Suter A, Eskelinen EL, Hartmann D, Lüllmann-Rauch R, et al Accumulation of autophagic vacuoles and cardiomyopathy in LAMP-2-deficient mice. Nature 2000; 406: 902–906. 10972293 10.1038/35022595

[35] Parra V, Eisner V, Chiong M, Criollo A, Moraga F, Garcia A, et al Changes in mitochondrial dynamics during ceramide-induced cardiomyocyte early apoptosis. Cardiovasc Res 2008; 77: 387–397. 18006463 10.1093/cvr/cvm029

[36] Zheng M, Xiao RP. Role of mitofusin 2 in cardiovascular oxidative injury. J Mol Med (Berl) 2010; 88: 987–991. 10.1007/s00109-010-0675-5 20824264

[37] Pich S, Bach D, Briones P, Liesa M, Camps M, Testar X, et al The Charcot-Marie-Tooth type 2A gene product, Mfn2, up-regulates fuel oxidation through expression of OXPHOS system. Hum Mol Genet 2005; 14: 1405–1415. 15829499 10.1093/hmg/ddi149

[38] Bach D, Pich S, Soriano FX, Vega N, Baumgartner B, Oriola J, et al Mitofusin-2 determines mitochondrial network architecture and mitochondrial metabolism. A novel regulatory mechanism altered in obesity. J Biol Chem 2003; 278: 17190–17197. 12598526 10.1074/jbc.M212754200

[39] Chen KH, Dasgupta A, Ding J, Indig FE, Ghosh P, Longo DL. Role of mitofusin 2 (Mfn2) in controlling cellular proliferation. Faseb j 2014; 28: 382–394. 10.1096/fj.13-230037 24081906 PMC3868832

[40] Bach D, Naon D, Pich S, Soriano FX, Vega N, Rieusset J, et al Expression of Mfn2, the Charcot-Marie-Tooth neuropathy type 2A gene, in human skeletal muscle: effects of type 2 diabetes, obesity, weight loss, and the regulatory role of tumor necrosis factor alpha and interleukin-6. Diabetes 2005; 54: 2685–2693. 16123358 10.2337/diabetes.54.9.2685

[41] Liu H, He Z, Simon HU. Autophagy suppresses melanoma tumorigenesis by inducing senescence. Autophagy 2014; 10: 372–373. 10.4161/auto.27163 24300435 PMC5396100

[42] Rabinowitz JD, White E. Autophagy and metabolism. Science 2010; 330: 1344–1348. 10.1126/science.1193497 21127245 PMC3010857

[43] Degenhardt K, Mathew R, Beaudoin B, Bray K, Anderson D, Chen G, et al Autophagy promotes tumor cell survival and restricts necrosis, inflammation, and tumorigenesis. Cancer Cell 2006; 10: 51–64. 16843265 10.1016/j.ccr.2006.06.001 PMC2857533

[44] Jin D, Zhang Y, Chen X. Lipocalin 2 deficiency inhibits cell proliferation, autophagy, and mitochondrial biogenesis in mouse embryonic cells. Mol Cell Biochem 2011; 351: 165–172. 10.1007/s11010-011-0724-6 21234651

