# Supplementary figures and images for: Mitofusin 2-Deficiency Suppresses Cell Proliferation through Disturbance of Autophagy
Source: PLoS One. 2015 Mar 17;10(3):e0121328. doi: 10.1371/journal.pone.0121328 (PMC4363693; doi:10.1371/journal.pone.0121328)

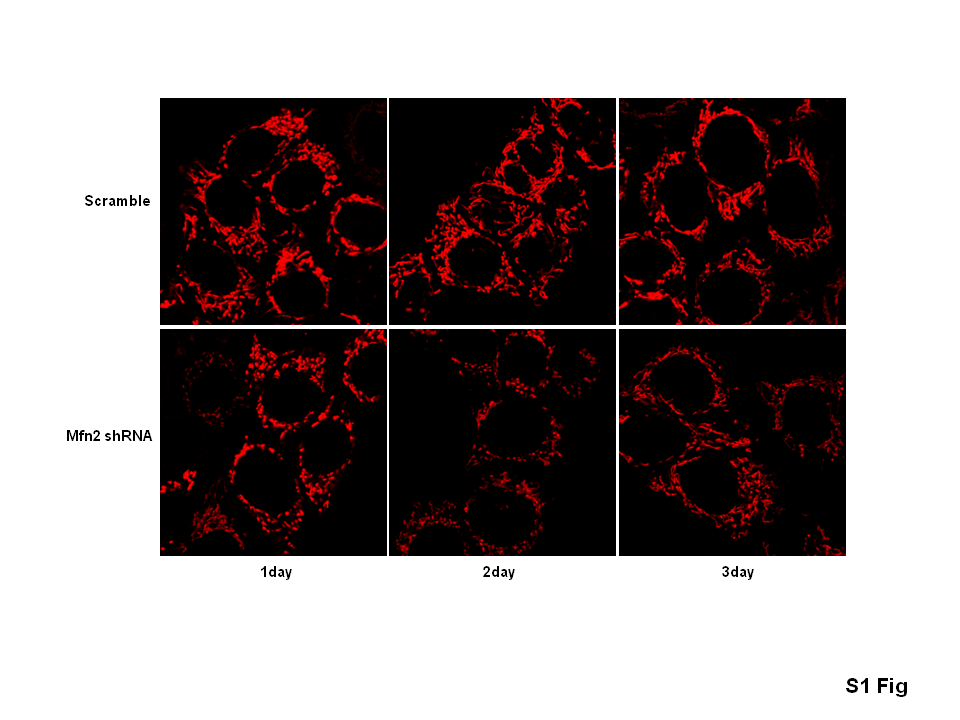

Supplement: S1 Fig — Confocal imagings of scramble and Mfn2 shRNA transfected HeLa cells stained with mitoTrackor showing mitochondrial morphology at time points after transfection as indicated. (TIF) [file pone.0121328.s001.TIF]

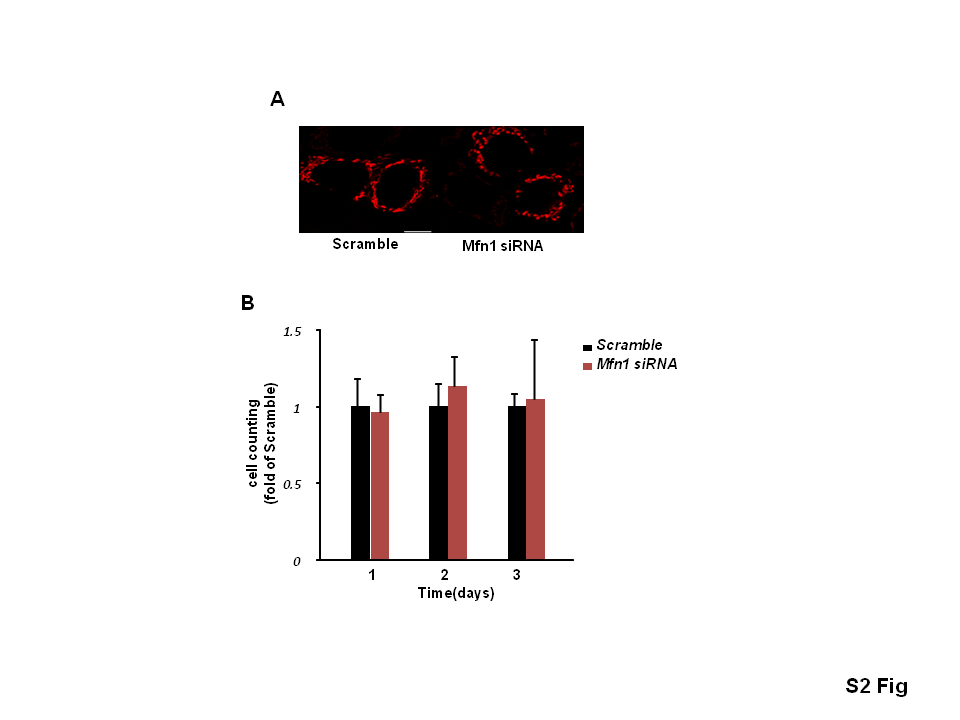

Supplement: S2 Fig — (A) Confocal imagings of scramble (left) and Mfn1 siRNA (right) infected HeLa cells stained with mitoTrackor. (B) Cell counting kit-8 (CCK8) assay of scramble or Mfn1 siRNA infected HeLa cells at indicated time after infection. (TIF) [file pone.0121328.s002.TIF]

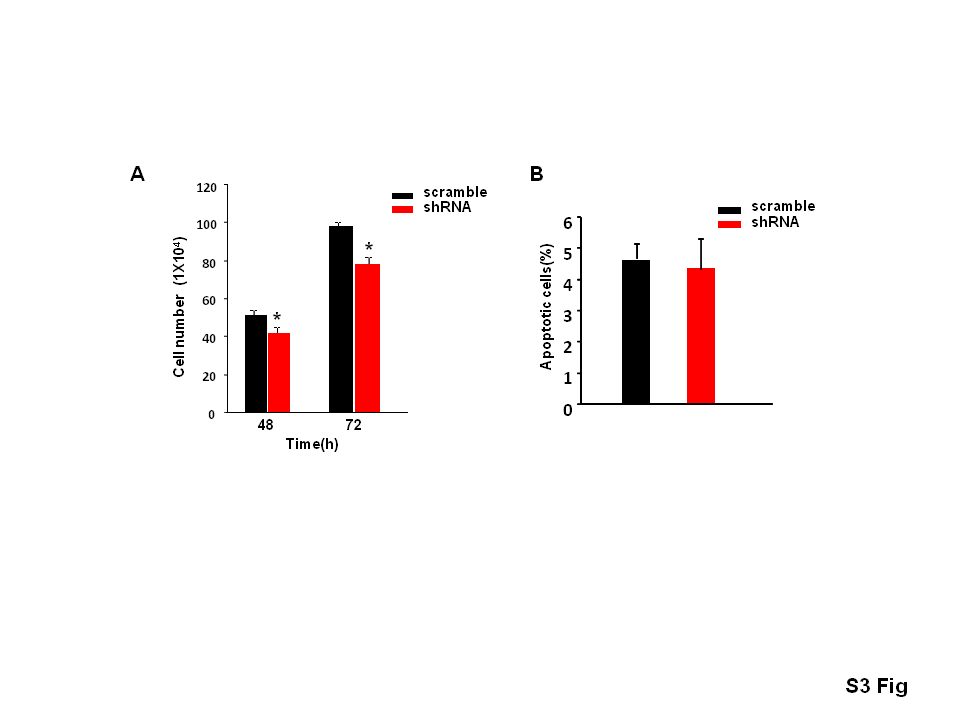

Supplement: S3 Fig — (A) Cell number counting of scramble and Mfn2 shRNA transfected HeLa cells by a cytometer at indicated time point after transfection. (B) Apoptotic cell counting of Hela cells stained with Hoechst 33342 by fluorescence microscope. (TIF) [file pone.0121328.s003.TIF]

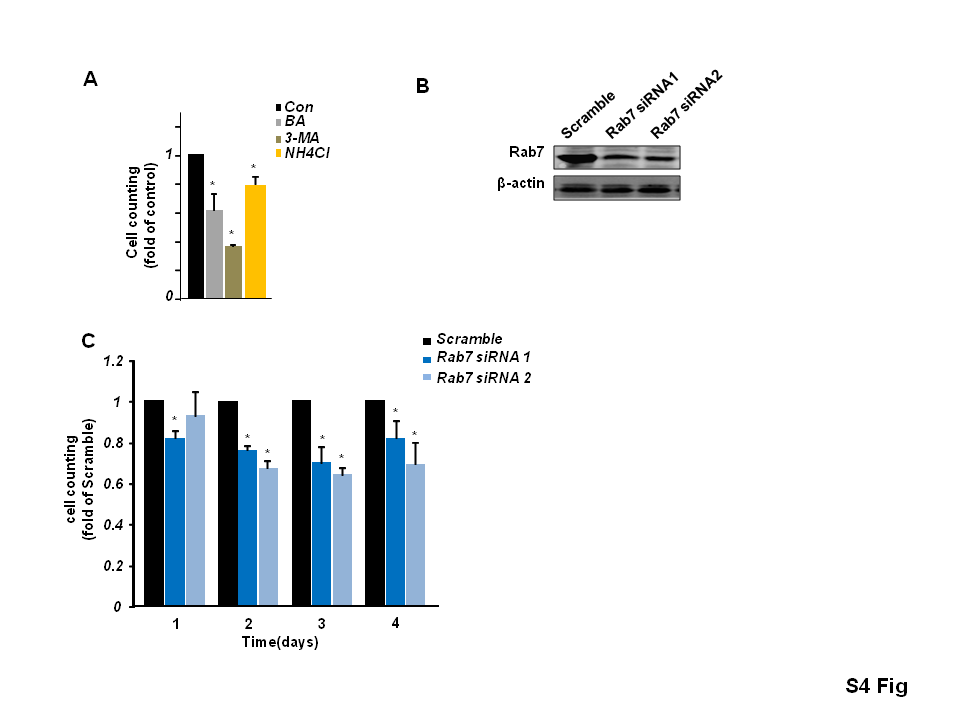

Supplement: S4 Fig — (A) Relative fold changes of cell counting by CCK8 in HeLa cell treated with autophagic degradation inhibitors Bafilomycin A1, 3-Methyladenine (3-MA), or NH4Cl as comparing with cells treated with DMSO. n = 3 independent experiments for each group. (B) Rab7 protein levels by western blotting in HeLa cells transfected with scrambled RNA, Rab7 siRNA1, or Rab7 siRNA2. n = 3 independent experiments. (C) Fold changes of cell counting by CCK8 in HeLa cells transfected with Rab7 siRNA1, or Rab7 siRNA2 comparing with cells transfected with scrambled RNA. n = 3–5 independent experiments. *, p<0.05 versus control. (TIF) [file pone.0121328.s004.TIF]

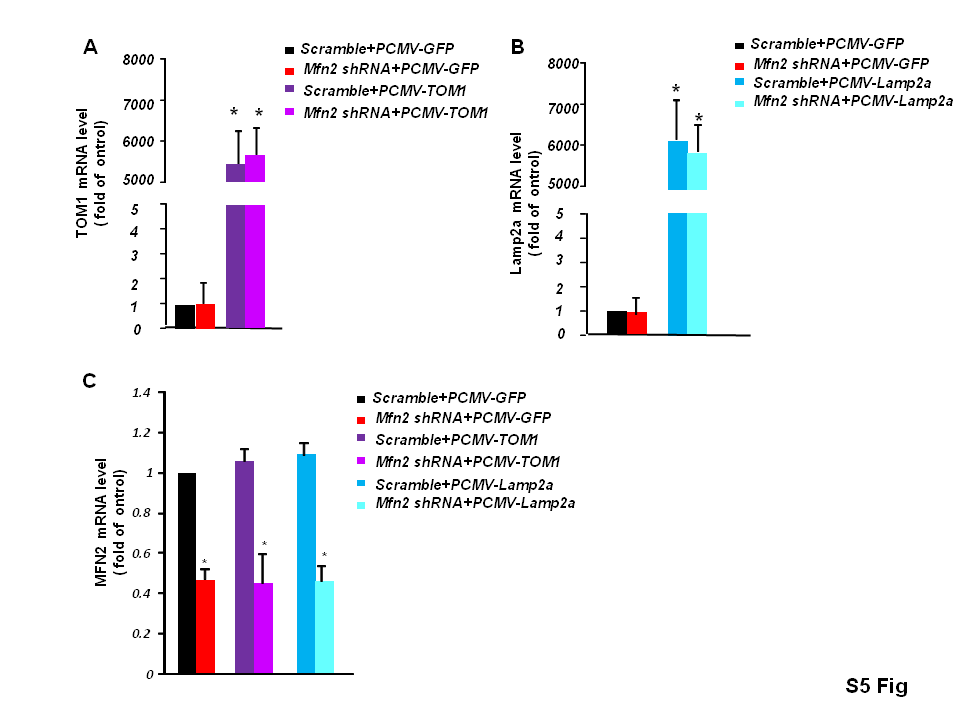

Supplement: S5 Fig — (A) mRNA level of Tom 1 by RT-PCR in scramble or Mfn2 shRNA transfected cells co-expressed with Pcmv-GFP or Pcmv-Tom1 plasmids. (B) mRNA level of Lamp2a by RT-PCR in scramble or Mfn2 shRNA transfected cells co-expressed with Pcmv-GFP or Pcmv-Lamp2a plasmids. (C) mRNA level of Mfn2 by RT-PCR in scramble or Mfn2 shRNA transfected cells co-expressed with Pcmv-GFP, Pcmv-Tom1, or Pcmv-Lamp2a plasmid. n = 3 independent experiments. (TIF) [file pone.0121328.s005.TIF]

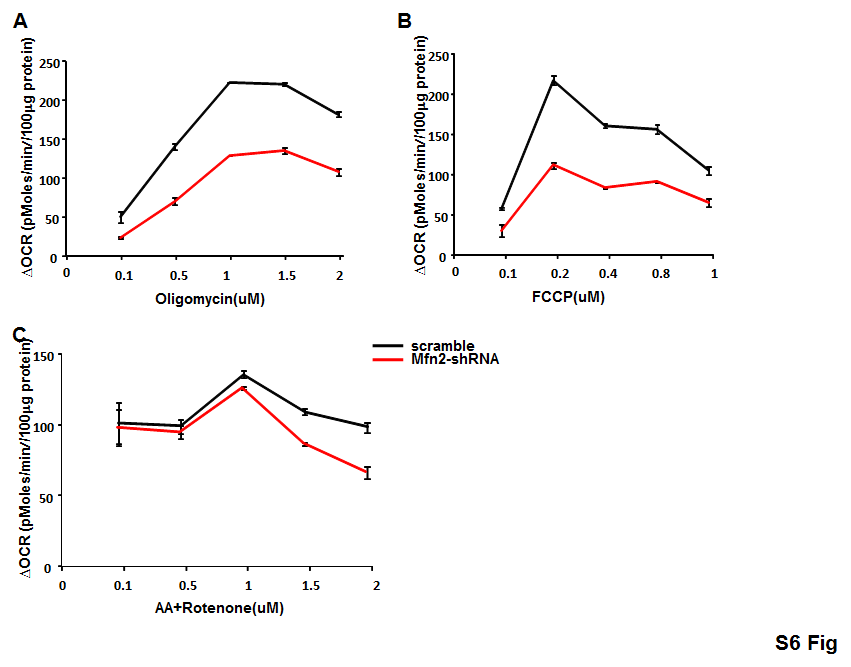

Supplement: S6 Fig — Data were presented as difference of OCR between cells with and without mitochondrial inhibitor stimulation. n = 3 independent experiments for each group. (TIF) [file pone.0121328.s006.tif]

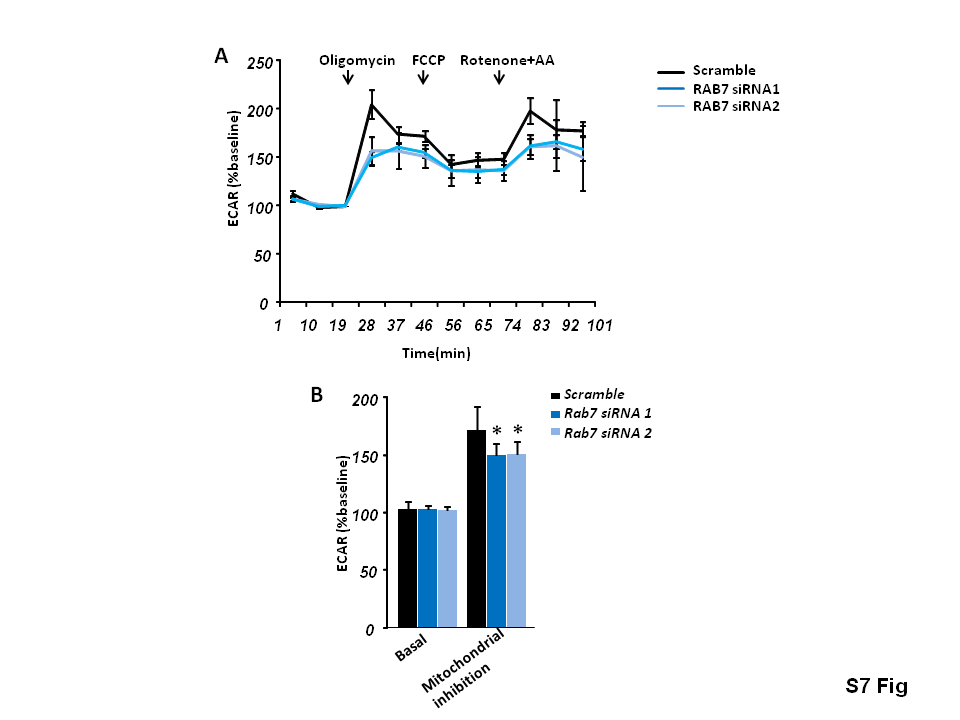

Supplement: S7 Fig — (A) Traces of extracellular acidification rates (ECAR) of HeLa cells in response to mitochondrial inhibitors. (B) Average data of basal and ECAR in the presence of mitochondrial inhibitors as in A. n = 3 independent experiments for each group. *, p<0.05 versus scramble control. (TIF) [file pone.0121328.s007.TIF]
